# PET Imaging of Neutrophils Infiltration in Alzheimer's Disease Transgenic Mice

**DOI:** 10.3389/fneur.2020.523798

**Published:** 2020-12-10

**Authors:** Yanyan Kong, Kawai Liu, Tao Hua, Chencheng Zhang, Bomin Sun, Yihui Guan

**Affiliations:** ^1^Department of Neurosurgery, Center for Functional Neurosurgery, Ruijin Hospital, Shanghai Jiao Tong University School of Medicine, Shanghai, China; ^2^PET Center, Huashan Hospital, Fudan University, Shanghai, China; ^3^Department of Mathematics, The Shanghai SMIC Private School, Shanghai, China

**Keywords:** neutrophils, Alzheimer's disease, PET, tau, Aβ, neuroinflammation

## Abstract

Neutrophils are important components in the innate immune system. Neutrophil hyperactivation is regarded as a characteristic of Alzheimer's disease (AD). But *in vivo* imaging tools observing neutrophil activity in AD dynamically is lacking. This study aimed to identify neutrophil infiltration in AD transgenic mice. We used the AD triple-mutant transgenic mouse model and identified the genotype with RT-PCR. Behavioral experiments including an open-field test, a Morris water maze, and a Y-maze test were performed to evaluate the status of this AD model. ^18^F-AV45, ^18^F-PM-PBB3, ^68^Ga-PEG-cFLFLFK, and ^18^F-DPA714 were synthesized according to previous reports. We employed microPET to detect tracer uptake in the AD model and the control mice at different stages. Western blotting was used to observe the expression of functional proteins. We proved the successful establishment of AD models by RT-PCR, behavioral tests, and ^18^F-AV45 and ^18^F-PM-PBB3 PET imaging. We found an increased neutrophil accumulation in the brains of the AD mice through ^68^Ga-PEG-cFLFLFK PET imaging and Western blot assay. Our studies also demonstrated an elevated level of CAP37, which is produced by neutrophils, in the AD brain, and treatment with CAP37 promoted the expression of Iba1, iNOS, and COX-2 in BV2 cultures. Furthermore, our ^18^F-DPA714 PET imaging studies verified the raised activation of microglia in the brain of transgenic AD mice. Collectively, our findings indicate the increased activity of neutrophils in the brain and heart of AD model mice, ^68^Ga-PEG-cFLFLFK PET imaging represents a sensitive method to observe the status of neutrophils in AD, and infiltrated neutrophils can induce the activation of microglia by releasing CAP37 and blocking the activity of neutrophils may be beneficial for the control of AD progression.

## Introduction

Alzheimer's disease (AD) is a neurodegenerative condition characterized by the formation of amyloid-β plaques, aggregated, hyper, and abnormally phosphorylated tau protein, activated microglia and neuronal cell death, ultimately leading to progressive dementia. AD is the most common cause of cognitive impairment or dementia in populations over 65 years old and, with rising longevity, a worldwide pandemic of mild cognitive impairment (MCI), AD, and AD-related dementia (ADRD) is anticipated (1–4). For many years, AD was viewed as a disease limited to the brain. Nevertheless, microglia is an innate immune cell in the brain, and brain-initiated inflammatory responses reflected in the periphery suggests that AD is to some extent also a systemic inflammatory disease (5). The activation status of peripheral innate immune cells may represent an early biomarker of the upcoming impact on the brain.

Neutrophils are among the first leukocytes to reach a site of injury and kill pathogens by various strategies, including phagocytosis, degranulation, the rapid production of reactive oxygen species (ROS) in an oxidative burst, the release of neutrophil extracellular traps (NETs), and a process called NETosis (6, 7). Several previous reports have shown the presence of neutrophils in the brain of AD patients, including in brain parenchyma with Aβ deposits and cerebral blood vessels (8–10). In the transgenic AD model mice brain, there is also substantial neutrophil infiltration to Aβ plaques, and neutrophil depletion or trafficking inhibition can reduce the AD-like neuropathology and improve the cognitive dysfunction (10, 11). Neutrophils can also damage an AD brain via NETosis that impairs the blood-brain barrier and neural cells in mouse AD models (10, 12). A recent *in vitro* study suggested that various proinflammatory cytokines directly increase the percentage of senescent neutrophils and decrease the level of immunosuppressive neutrophils, the peripheral proinflammatory cytokine environment is instrumental in the alteration of circulating neutrophils homeostasis in AD (7). In consequence, the ability to detect and quantify neutrophilic accumulation could be important not only in locating and identifying inflammatory lesions, but also in facilitating the determination of the pathological development and testing of anti-inflammatory agents in AD patients (13). A previous study investigated the expression of the cationic antimicrobial protein of 37 kDa (CAP37), a neutrophil granule protein, in AD, and demonstrated an upregulation of CAP37 in patients with AD (9, 14).

Neuroimaging modalities, such as positron emission tomography (PET), optical scanning, and magnetic resonance imaging (MRI), have enabled the visualization of Aβ deposits in humans with AD or AD mouse models (15). Currently, ^67^Ga citrate and ^111^In or ^99m^Tc labeled leukocytes *ex vivo* are available clinical nuclear imaging probes for targeting and diagnosing inflammatory lesions (16). Unfortunately, each of these tracers possesses significant drawbacks even though they may yield useful results in specific circumstances. Modalities utilizing the *in vitro* labeling of white blood cells also suffer from certain disadvantages. In contrast, the injection of peptides, that have a high affinity for surface receptors on leukocytes, have emerged as an attractive option for the *in vivo* detection of inflammation. *In vitro* characterization of ^18^F-AV-45 and ^18^F-DPA-714 reported an excellent affinity and specificity for Aβ plaques (17, 18). Furthermore, ^18^F-PM-PBB3 was developed as a specific imaging ligand to visualize tau pathologies in the brains of patients with AD and related tauopathies in a tauopathy mouse model (15, 19).

The coexistence of reliable PET imaging methods with dedicated tracers in small animals and relevant animal models of AD is an opportunity to better understand the pathophysiological mechanisms of the disease and to monitor potential therapeutic approaches. The aim of this study was 2-fold: to characterize *in vivo* the phenotypes and functionalities of microglial cells in an AD animal model, and to explore the mechanisms of neuroinflammation in the onset and progression of AD.

## Materials and Methods

### Animals

The animal experimental protocol was approved by the Institutional Animal Care and Ethics Committee of Huashan Hospital of Fudan University. Animal experiment procedures were performed in strict accordance with the National Research Council's Guide for the Care and Use of Laboratory Animals. The AD triple-mutant transgenic (TG) model mice [B6;129-Psen1tm1MpmTg(APPSwe,tauP301L)1Lfa/Mmjax] and female wild-type mice (same background) were purchased from the Jackson Laboratory. The mean bodyweight of the mice was (20 ± 3) g, and the age of the animals when used for the experiment was 12-month-old. The characteristics and quality of the TG model mice were evaluated by behavioral experiments, such as an open field test, a Morris water maze, and a Y-maze test.

### Tracer Production

^18^F-AV-45 was radio synthesized from its precursor (Avid, USA) in a fully automated procedure suitable for routine clinical application (20). ^18^F-PM-PBB3 was synthesized from an automatic synthesis module and kit provided by APRINDIA therapeutics (Suzhou, China) (21). The formyl peptide receptor (FPR)-specific peptide, PEG-cFLFLFK, was sequentially conjugated with a DOTA, and finally labeled with ^68^Ga to form the ^68^Ga-cFLFLFK tracer (13). DPA-714 was labeled with ^18^F at its 2-fluoroethyl moiety, after nucleophilic substitution of the corresponding tosylate analog, according to a previously reported procedure with slight modifications (22). The radiochemical purity of the final purified products all exceeded 95%.

### PET Imaging and Data Analysis

Mice were anesthetized with isoflurane and intravenously injected with ^18^F-AV45 (100 ± 20 μCi), ^18^F-PM-PBB3 (100 ± 20 μCi), ^68^Ga-PEG-cFLFLFK (100 ± 20 μCi) (or unlabeled cFLFLFK peptide for blocking FPR1 receptors), and ^18^F- DPA714 (100 ± 20 μCi) via the lateral tail vein in different groups. Every mouse in each group only received one type of tracer injection and PET imaging scans. The 15 min static images were obtained by small-animal PET/CT systems (at 1 h after injection), and scanned at 350–650 keV energy window, with 20 min listmode acquisition and 3D rebinning followed by OSEM-PSF reconstruction. PET images were analyzed and quantified by PMOD software and data were calculated in 3D volumes-of-interest (VOIs) using the following equation. The VOIs or ROIs (regions of interest) were delineated by manual selection of the statistical investigator blind to experimental design and grouping in the reconstructed 3D images. After delineation, the radioactivity values of the ROIs per unit volume were obtained, and the percentage injected dose per gram (%ID/g) values were calculated.

$$\begin{array}{l} \%\text{ID}/\text{g}=\frac{\text{Radioactivity concentration in VOIs }\left[\text{μCi}/\text{g}\right]}{\text{Injected dose }\left[\text{μCi}\right]}\times 100\text{\%} \end{array}$$

### Western Blot Assay

The procedures of western bolt are the same as the previous references (23). Simply, the mice were euthanized in 30 min after the last PET scan, and the brains from the AD transgenic and control mice were dissected and lysed in animal tissue lysis buffer. Then, the mixtures were centrifuged at 4°C and 10,000 g for 15 min, and the supernatants were separated as total protein samples for Western blot assays. As to the microglia BV2 cell culture, rCAP37 protein (1 μg/mL, final concentration in culture medium) was applied and treated for 24 h according to a previous report (24), and the cells was harvested for Western blotting. A portion of the sample with 20 μg protein was loaded in SDS-PAGEs. For our Western blot assays, primary antibodies for mouse FPR1 (1:1,000), Iba1 (1:500), CAP37 (1:200), iNOS (1:1,000), and COX-2 (1:1,000), and secondary antibodies were purchased from Abcam.

### Immunohistochemistry Assay

The transgenic AD model and WT control mice were anesthetized and rapidly perfused and fixed with 4% paraformaldehyde. The brains were removed and placed in 4% paraformaldehyde for 24 h, and after dehydration in 30% sucrose, sectioned into slices 20 μm thick. After antigen repair and blocking, the slices were incubated with an anti-myeloperoxidase (MPO) antibody (1:1,000) at 4°C and gently oscillated overnight. Following second antibody incubation and wash, the nucleus was stained with hematoxylin. Then after DAB color rendering and sealing, the slices were observed under a microscope (Olympus BX43, Japan), and the images were captured and stored. The integral optical density (IOD) of the MPO stains of neutrophils per area was quantified with the Scion Image 4.0 software (Scion Inc., Fredrick, MD).

### Statistical Analysis

Numerical data are presented as mean ± SEM. The Student's unpaired *t*-test (for normal distribution) or Mann-Whitney U-test (for non-normal distribution) were used to analyze the differences between groups, including the results of the VOIs radioactive uptake, and a difference at *p* < 0.05 was considered statistically significant.

## Results

### Generation and Characterization of AD Model Mice

**Figure 1:**
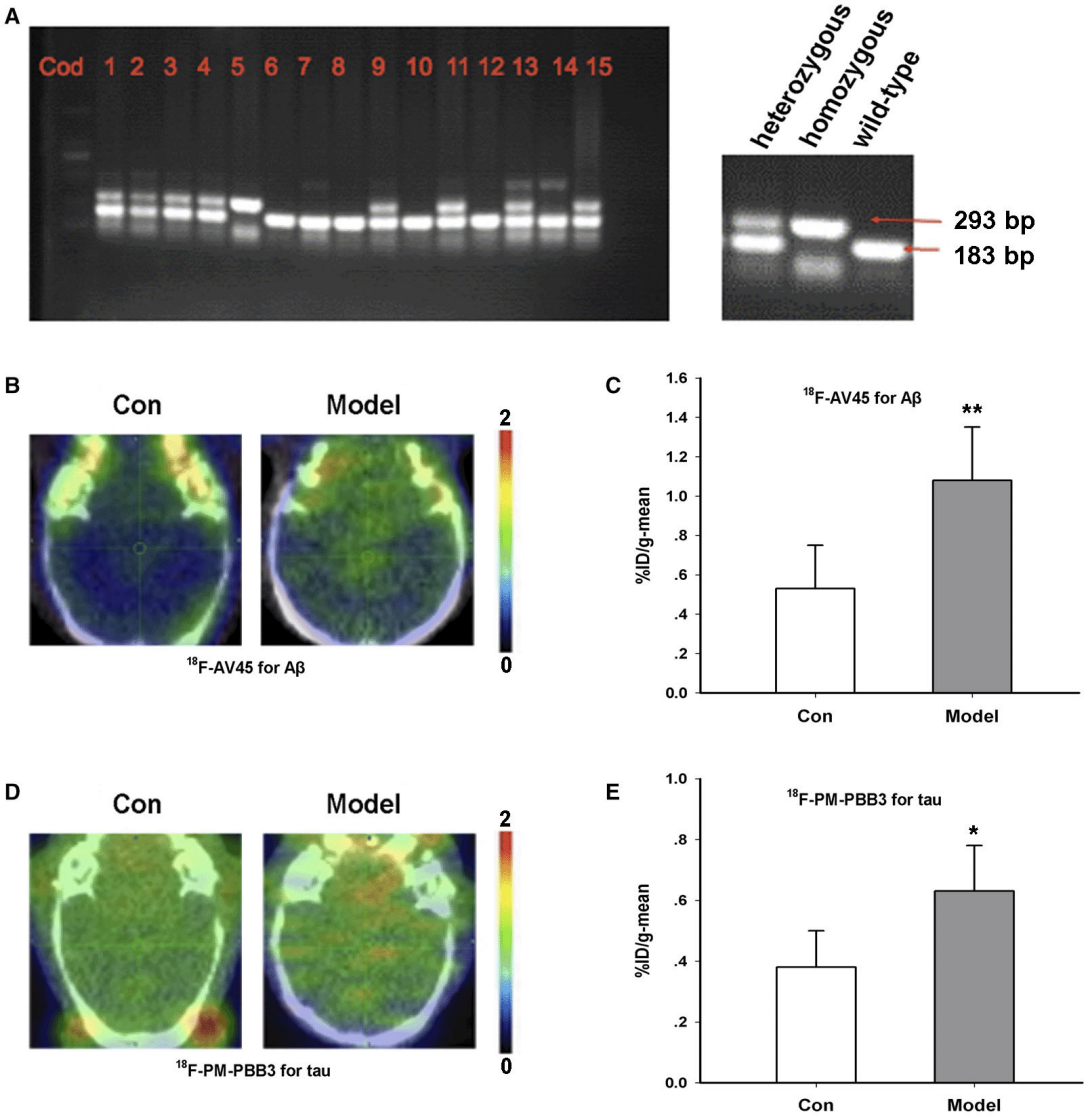
Characterization of AD transgenic animal model. **(A)** Identification of gene expression by RT-PCR. **(B)** ^18^F-AV45 imaging in the brain of the TG model and control mice. **(C)** Radioactive uptake of ^18^F-AV45 in the brain of mice *in vivo*. **P = 0.009, *n* = 5 mice for each group. **(D)** ^18^F-PM-PBB3 imaging in the brain of the model and control mice. **(E)** Radioactive uptake of ^18^F- PM-PBB3 in the brain of mice *in vivo*. *P = 0.012, *n* = 5 mice for each group. This part of content has been published as a conference abstract in the Annual Congress of the European Association of Nuclear Medicine, October 12–16, 2019, Barcelona, Spain.

The AD transgenic model mice (TG/model) and female wild-type mice (WT/control) were purchased from the Jackson Laboratory. We used RT-PCR to identify all the experimental animals in this study as to determine whether they were homozygous or heterozygous (Figure 1A). The content corresponding to Figure 1 has been published as a conference abstract in the Annual Congress of the European Association of Nuclear Medicine, October 12–16, 2019, Barcelona, Spain (25). Based on the literature, the animal model only produced amyloid in 6 months (26). Hence, we began the observation of animal behaviors from 6 months of age. At the age of 12 months, most of the TG model group exhibited behavioral abnormalities (Supplementary Figure 1).

^18^F-AV45 is a classic PET imaging tracer for Aβ and is often used to detect Aβ aggregation in preclinical and clinical studies (27). Furthermore, ^18^F-PM-PBB3 is a new type of tau tracer for PET imaging (28). In order to further confirm the animal model, we used these two tracers to identify the 12-month-old TG model of AD. As shown in Figures 1B–E, compared with the control group, the %ID/g-mean of ^18^F-AV45 in the TG model was significantly higher than that of control (Con, 0.53 ± 0.22; Model, 1.08 ± 0.27, *p* = 0.009 vs. Con) (Figures 1B,C). The uptake of ^18^F-PM-PBB3 in the TG group was significantly higher than that in the control group (Con, 0.38 ± 0.12; Model, 0.63 ± 0.15, *p* = 0.012 vs. Con) (Figures 1D,E).

Taken together, we identified and confirmed the success of the AD model according to RT-PCR, behavioral tests, and the results of the ^18^F-AV45 and ^18^F-PM-PBB3 PET imaging.

### Increased Neutrophil Infiltration in the AD Animal Model

**Figure 2:**
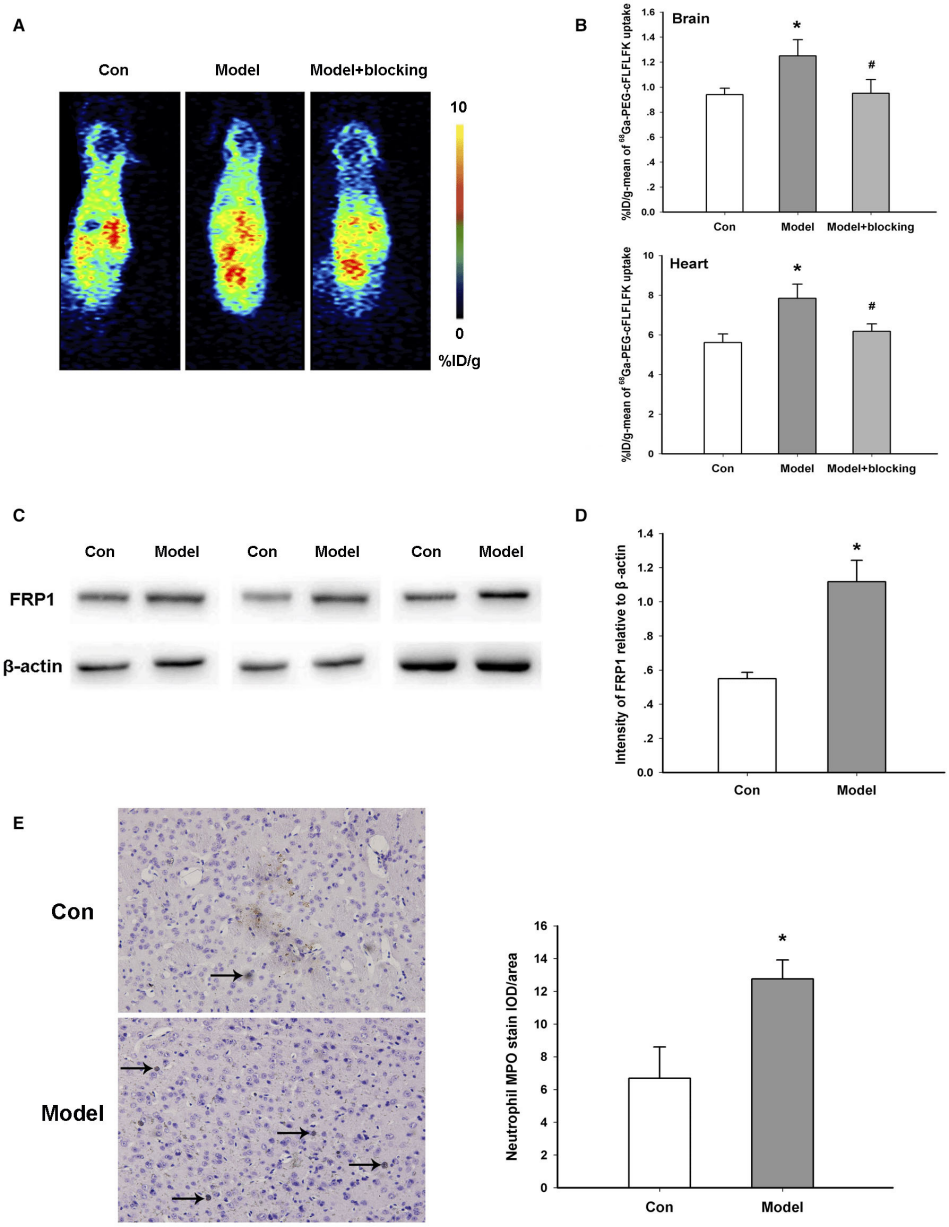
Increased neutrophil infiltration in the transgenic animal model. **(A)** Representative ^68^Ga-PEG-cFLFLFK PET imaging in the brain of the model and control mice. **(B)** Radioactive uptake of ^68^Ga-PEG-cFLFLFK in the brain and heart of mice *in vivo*. Brain, *p = 0.036 for model vs. Con, #*p* = 0.043 for model+blocking vs. model, *n* = 5 mice for each group; Heart, *p = 0.042 for model vs. Con, #*p* = 0.047 for model + blocking vs. model, *n* = 5 mice for each group. **(C)** Western blot study showing the increase in FRP1 expression in the brain of AD model mice. **(D)** Quantification of the protein blot density of FRP1 in control and model mice. *P = 0.023, *n* = 5 mice for each group. **(E)** Representative results of immunohistochemistry assay with an antibody against the MPO protein. *P = 0.028, *n* = 10 areas from three mice for each group.

In order to evaluate the infiltration of neutrophils in the brain, we used both ^68^Ga-PEG-cFLFLFK PET imaging and Western blotting methods. ^68^Ga-PEG-cFLFLFK is a recently developed specific tracer for the FPR1 receptor on neutrophils (29). PET images scanned 60 min after tracer injection indicated that the %ID/g-mean of the brain and some other organs, especially the heart, was higher in the AD model group than the control group, and by blocking FPR1 receptors with the unlabeled cFLFLFK peptide, the increased radioactive uptake in the model group obviously diminished (Figures 2A,B). We also analyzed the radioactive uptake (%ID/g-mean and %ID/g-max) of multiple brain regions in the TG and WT groups (data of 14 brain regions are shown in Supplementary Table 1). Furthermore, the results of Western blotting showed that the expression of the FPR1 protein in the brain of AD model mice was significantly higher than that in the control group (Figures 2C,D).

MPO is a hemoprotein which is abundantly expressed in neutrophils and secreted during their activation. Results of an immunohistochemistry assay with an anti-MPO antibody show that the amount of neutrophils was significantly augmented in the TG mice of the AD model compared with the control (Figure 2E). In general, these results confirmed the infiltration of neutrophils in the brain of AD transgenic mice.

### Microglia Activation Induced by Neutrophils

**Figure 3:**
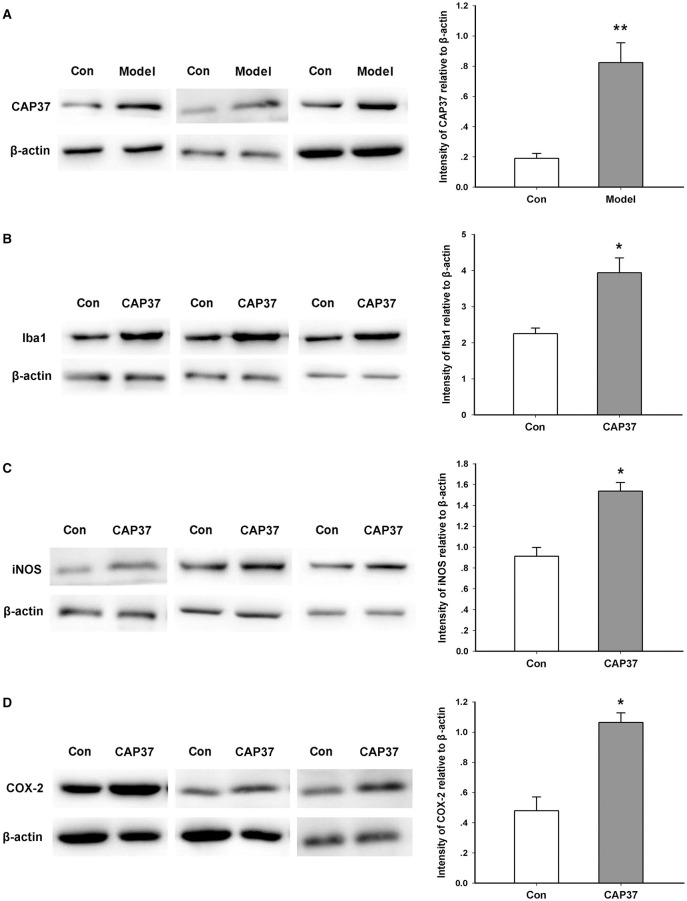
Activation of microglia induced by CAP37. **(A)** Western blot study showing the increase in CAP37 expression in the brain of AD model mice. **P = 0.006, *n* = 5 mice for each group. **(B)** Western blot study showing the increase in Iba1 expression in cultured microglia induced by CAP37. *P = 0.014, *n* = 5 for each group. **(C)** Western blotting study showing the increase in iNOS expression in cultured microglia induced by CAP37. *P = 0.027, *n* = 5 for each group. **(D)** Western blot study showing the increase in COX expression in cultured microglia induced by CAP37. *P = 0.019, *n* = 5 for each group.

In order to investigate the significance of neutrophil infiltration in the brain, we focused on the CAP37 protein and microglia. CAP37, a specific protein secreted by neutrophils, is a potential immune regulator in the brain. Consistent with previous studies (9), we found that the expression of CAP37 in the TG model group was significantly higher than that of the control group (Figure 3A). This result also confirmed the infiltration of neutrophils in the brain.

Iba1 is a marker of activated microglia which are the most important immune cells in the brain (30). As shown in Figure 3B, we found that exogenous administration of the CAP37 protein gave rise to an increased expression of the Iba1 protein in the microglia BV2 cell line *in vitro*. In addition, the expression levels of iNOS and COX-2 were also notably upregulated by CAP37 treatment (Figures 3C,D).

**Figure 4:**
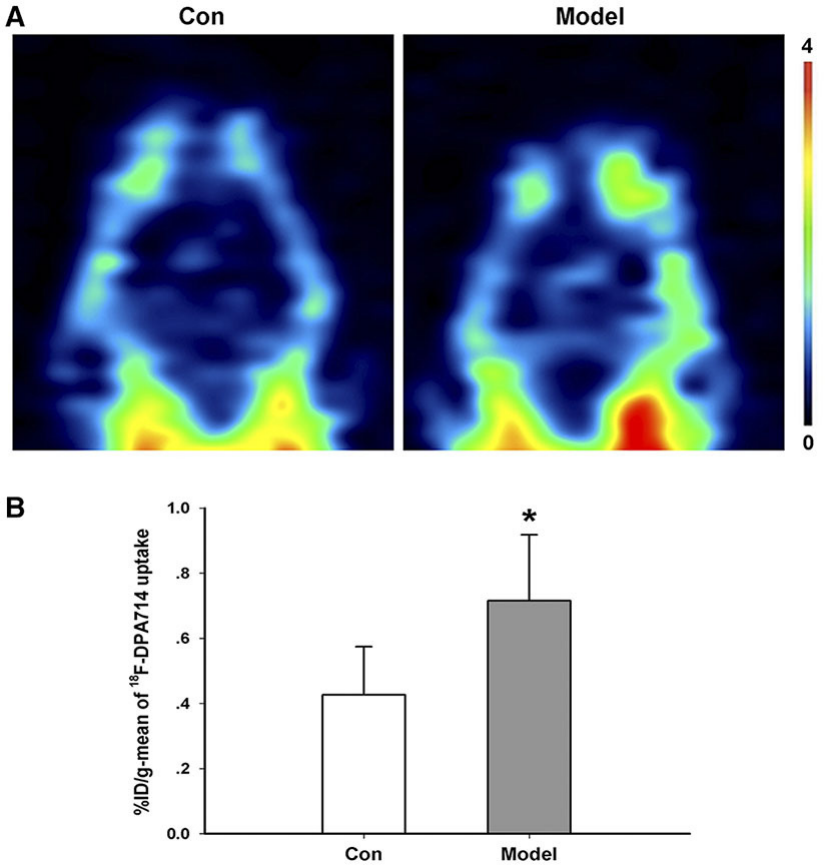
Increased microglia activation in AD transgenic model. **(A)** Representative ^18^F-DPA714 PET imaging in the brain of AD transgenic model and control mice. **(B)** Quantification of the radioactive uptake of ^18^F-DPA714 in the brain of the control and model groups. *P = 0.031, *n* = 5 mice for each group.

In order to study the dynamic alterations of microglia *in vivo*, we conducted an ^18^F-DPA714 PET imaging study. In the control group, the brain uptake of ^18^F-DPA714 was relatively low. Nevertheless, the radioactive uptake of ^18^F-DPA714 in the brain of the AD model mice increased significantly compared with the control group (%ID/g-mean) (Figures 4A,B).

## Discussion

Several articles have reported the relationship between the innate immune system and AD (31). Indeed, microglia are the typical innate immune cells in the central nervous system, while neutrophils are a representative of peripheral innate immune cells (32). However, the exact connection between the central and peripheral innate immune cells in the pathophysiology of AD remains elusive. In this study, we tried to uncover the underlying mechanism using several molecular imaging tracers *in vivo*.

Firstly, we identified AD transgenic models with several experiments, including RT-PCR, behavior tests, and ^18^F-AV45 and ^18^F-PM-PBB3 PET imaging. To the best of our knowledge, we are the first to confirm a triple transgenic model by Aβ and tau tracer PET imaging *in vivo* at the same time. Secondly, we proved the neutrophil infiltration in the brain of TG mice by a specific neutrophil PET tracer *in vivo* and *ex vivo*. Additionally, our study demonstrated that CAP37 is an important modulator in microglia activation, which indicates that neutrophil infiltration promotes the neuroinflammation of AD.

In this study, we selected triple transgenic AD mice, which were classical models for studying amyloid deposition, tau pathology, and synaptic transmission. These mice expressed three mutant genes: Psen1 M146V, APPSwe, and Tau P301L (26). Generally, we identified them through immunohistochemistry and behavioral tests. Molecular imaging, such as PET and SPECT, is a very powerful tool for the diagnosis of Parkinson's disease (33, 34). However, most preclinical studies are based on tracers for Aβ or tau separately (15, 35). In this study, we could better observe the pathophysiological process of the animal using the two tracers together. Amyloid deposits and tau fibrils are also typical characteristics of the model. With ^18^F-AV45 PET imaging, we found that radioactive uptake is relative higher in the brain of TG mice compared with the control group. We also observed more serious tau fibrosis in the same model using ^18^F-PM-PBB3 PET imaging.

Numerous clinical trials have confirmed the high activation of neutrophils in the peripheral blood of AD patients. Normally, there is limited infiltration of neutrophils in the brain. There is also a lack of direct imaging evidence to study the neutrophil infiltration in AD patients. cFLFLFK is a high affinity ligand for N-formylpeptide receptor 1 (FPR1), which is highly expressed in peripheral neutrophils. Previous studies have proved the application of ^68^Ga-PEG-cFLFLFK in neutrophils imaging (29). In this study, we verified the neutrophil infiltration by ^68^Ga-PEG-cFLFLFK and Western blotting. Then we hypothesized that neutrophil infiltration, secretion proteins, and inflammatory factors accelerate the activation of microglia, the neuroinflammatory response, tau protein lesions, and Aβ deposition of starch protein in the process of AD.

CAP37, originating from neutrophils, is a critical inflammatory modulator in the immune system. Some scholars have found the increased expression of CAP37 in AD mice (9, 14). Consistent with these studies, we also observed that the expression of CAP37 in the brain of AD transgenic mice was significantly higher than control. Furthermore, our results demonstrated that the administration of CAP37 promoted cultured microglia activation. We also confirmed the hyper-activation of microglia in the AD model by ^18^F-DPA714, a specific tracer targeting translocate protein in activated microglia.

In AD patients and animal models, in addition to directly interfering with the normal activity and function of neurons, Aβ acts as a potent chemoattractant to induce the migration of phagocytes, including neutrophils and macrophages, into the brain, causing and maintaining neuroinflammation (36, 37). The early presence of Aβ in the AD brain activates microglia and neutrophils and promotes the release of masses of toxic mediators including inflammatory cytokines and ROS, leading to the maintenance and exacerbation of harmful pathological inflammatory responses, such as cytokine storms, which mediates progressive neural damage and loss of function (38–41). Neutrophils can also damage the blood-brain barrier and lead to increased permeability through NETosis (12). Some studies, including PET imaging, indicate that there are neuroinflammation and microglia activation in the early stages of AD (40–42). However, there is no direct imaging evidence of neutrophil activation in an AD brain. This study, the first to use PET tracer imaging in an AD animal model, showed increased neutrophil invasion and activation in the brain of the AD model mice. At the same time, the neutrophil constitutive granule protein CAP37 mediates the hyper-activation of microglia in the AD brain. Thus, neuroinflammatory reactions mediated by neutrophils and microglia have a co-driving role in the maintenance of progressive inflammatory brain injury in AD, and neutrophil depletion and trafficking inhibition may represent a helpful intervention to occlude neuroinflammation and improve cognitive function in AD patients.

## Conclusion

In summary, we found that the combination of ^18^F-AV45 and ^18^F-PM-PBB3 tracers can be used to identify the triple-transgenic AD animal models. ^68^Ga-PEG-cFLFLFK is confirmed to be an ideal PET tracer to investigate the behavior and mechanism of neutrophils in central nervous system diseases *in vivo*. In addition, CAP37 may represent an important link between neutrophil infiltration and microglia activation in AD, and be of potential importance in driving and sustaining neuroinflammation and dysfunctions. Therefore, inhibiting neutrophil infiltration and activation may be beneficial for the amelioration of neuroinflammation and the control of AD symptoms.

## Data Availability Statement

All datasets generated for this study are included in the article/Supplementary Material.

## Ethics Statement

The animal protocol was approved by Institutional Animal Care and Ethics Committee of Huashan Hospital of Fudan University.

## Author's Note

The corresponding data of Figure 1 of this article have been published as a conference abstract in the Annual Congress of the European Association of Nuclear Medicine, October 12–16, 2019, Barcelona, Spain. The authors of the published conference abstract and this article all agree on the reuse and citation of this part of the content, and they thank the copyright holder (EANM) for permission to cite and reuse the material.

## Author Contributions

YG, BS, and CZ conceived and designed this study. YK and KL were responsible for the collection, extraction, and analysis of the data. YK was responsible for writing the paper. CZ, BS, YG, and TH polished the English language. All authors and participants reviewed the paper and reached an agreement to approve the final manuscript.

## Conflict of Interest

The authors declare that the research was conducted in the absence of any commercial or financial relationships that could be construed as a potential conflict of interest.

## Supplementary Material

Supplementary Figure 1 Behavioral experimental test results for the verification of AD transgenic mouse model. **(A)** Open field test result for wild-type (WT) and AD transgenic model (TG) mice. **(B)** Y-maze test result for WT and TG mice. **(C)** Time spent in the target area for WT and TG mice in the Morris water maze test. **(D)** Number of times crossing the target area for WT and TG mice in the Morris water mase test. *N* = 5 mice for each group in every test. ^*^*p* < 0.05, ^**^*p* < 0.01, ^***^*p* < 0.001 vs. WT group. #*p* < 0.05, ##*p* < 0.01 vs. TG group.

Supplementary Table 1 Radioactive uptake values of ^68^Ga-PEG-cFLFLFK targeting neutrophils in the brain scanned at 1 h after injection and comparisons between the TG model and WT groups (*n* = 5 mice for each group).
