# Supplementary figures and images for: PET Imaging of Neutrophils Infiltration in Alzheimer's Disease Transgenic Mice
Source: Front Neurol. 2020 Dec 10;11:523798. doi: 10.3389/fneur.2020.523798 (PMC7758535; doi:10.3389/fneur.2020.523798)

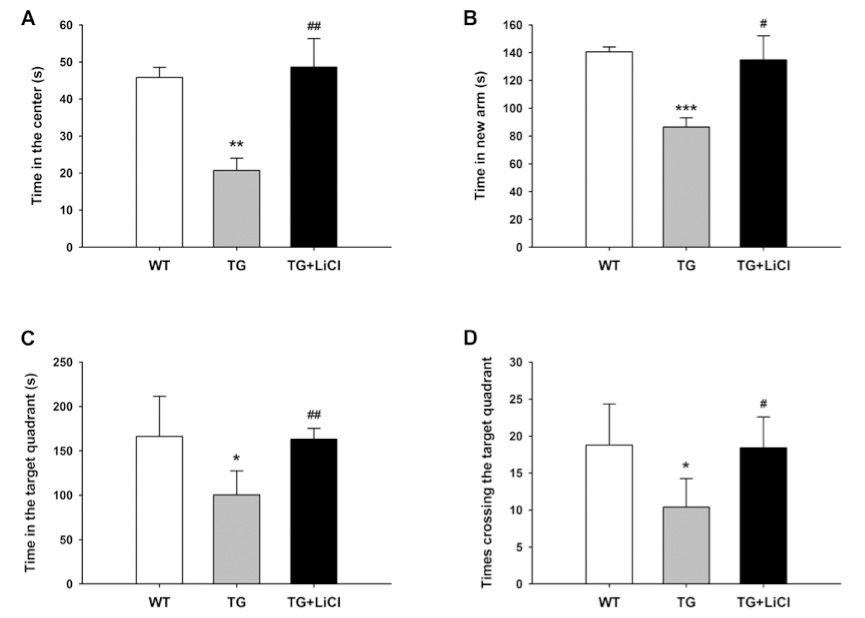

Supplement: Supplementary Figure 1 — Behavioral experimental test results for the verification of AD transgenic mouse model. (A) Open field test result for wild-type (WT) and AD transgenic model (TG) mice. (B) Y-maze test result for WT and TG mice. (C) Time spent in the target area for WT and TG mice in the Morris water maze test. (D) Number of times crossing the target area for WT and TG mice in the Morris water mase test. N = 5 mice for each group in every test. *p < 0.05, **p < 0.01, ***p < 0.001 vs. WT group. #p < 0.05, ##p < 0.01 vs. TG group. [file Image_1.JPEG]
