# Supplementary material for: PET Imaging of Neutrophils Infiltration in Alzheimer's Disease Transgenic Mice
Source: Front Neurol. 2020 Dec 10;11:523798. doi: 10.3389/fneur.2020.523798 (PMC7758535; doi:10.3389/fneur.2020.523798)
Supplement: Supplementary Table 1 — Radioactive uptake values of 68Ga-PEG-cFLFLFK targeting neutrophils in the brain scanned at 1 h after injection and comparisons between the TG model and WT groups (n = 5 mice for each group). [file Table_1.docx]

**Supplementary table**

**S Table 1** Radioactive uptake values of ^68^Ga-PEG-cFLFLFK targeting neutrophils in the brain scanned at 1 h after injection and comparisons between TG model and WT groups (n = 5 mice for each group)

| **Brain region** | **%ID/g-mean** | | | **%ID/g-max** | | |
| --- | --- | --- | --- | --- | --- | --- |
|  | **TG** | **WT** | **p value** | **TG** | **WT** | **p value** |
| Cortex | 1.40±0.26 | 0.77±0.16 | 0.003 | 3.18±0.74 | 1.98±0.21 | 0.01 |
| Thalamus | 1.00±0.33 | 0.61±0.10 | 0.038 | 2.16±0.66 | 1.27±0.30 | 0.03 |
| Cerebellum | 1.46±0.29 | 0.79±0.15 | 0.003 | 2.80±0.57 | 1.65±0.17 | 0.003 |
| Basal forebrain septum | 1.73±0.45 | 0.93±0.18 | 0.008 | 3.20±0.58 | 1.83±0.22 | 0.002 |
| Hypothalamus | 1.85±0.58 | 1.04±0.31 | 0.031 | 2.82±0.81 | 1.94±0.28 | 0.055 |
| Brain stem | 1.60±0.54 | 0.87±0.10 | 0.02 | 3.08±0.85 | 1.89±0.24 | 0.019 |
| Central gray | 0.94±0.45 | 0.71±0.45 | 0.325 | 1.51±0.40 | 1.26±0.26 | 0.294 |
| Superior colliculi | 1.06±0.29 | 0.66±0.13 | 0.028 | 1.80±0.11 | 1.10±0.21 | 0.0005 |
| Olfactory bulb | 1.72±0.31 | 1.04±0.16 | 0.003 | 2.97±0.90 | 1.94±0.32 | 0.048 |
| Striatum | 1.33±0.34 | 0.74±0.11 | 0.008 | 2.74±0.59 | 1.64±0.35 | 0.01 |
| Hippocampus | 1.24±0.26 | 0.71±0.13 | 0.006 | 2.52±0.95 | 1.41±0.29 | 0.04 |
| Amygdale | 1.93±0.64 | 1.03±0.24 | 0.022 | 2.90±0.78 | 1.69±0.28 | 0.014 |
| Midbrain | 1.17±0.28 | 0.65±0.15 | 0.009 | 2.67±0.90 | 1.21±0.20 | 0.009 |
| Inferior colliculi | 1.20±0.09 | 0.74±0.14 | 0.001 | 1.85±0.21 | 1.20±0.33 | 0.011 |
